# Stimulating appearance comparison dynamics and their effects on psychological dysfunctions: The moderating role of self-compassion

**DOI:** 10.1371/journal.pone.0293798

**Published:** 2023-11-09

**Authors:** Humma Nawaz, Mahwish Rabia, Hubba Javed, Muhammad Yousaf, Shahid Mahmood, Muhammad Riaz

**Affiliations:** 1 Department of Statistics, GC Women University, Sialkot, Sialkot, Pakistan; 2 Department of Statistics, Quaid-i-Azam University, Islamabad, Islamabad, Pakistan; 3 Government Degree College Batkhela, Batkhela, Malakand, Khyber Pakhtunkhwa, Pakistan; 4 Govt Murray Graduate College Sialkot, Sialkot, Pakistan; 5 Centre for Trials Research College of Biomedical & Life Sciences, Cardiff University, Cardiff, United Kingdom; Polytechnic Institute of Coimbra: Instituto Politecnico de Coimbra, PORTUGAL

## Abstract

In recent decades, attitudes towards appearance comparison, and self-disapproval have rapidly increased, and these are attitudes strongly associated with psychological disorders. The present study aims to investigate the underlying patterns of depression, appearance-based stress, dietary constraints, and social and celebrity appearance comparison among young adults. It also examines the role of self-compassion in moderating the relationship between psychological dysfunctions and appearance comparison as well as the criteria and influences contributing to appearance comparison. Data on BMI, the measures of depression, appearance-based stress, eating restraints, appearance comparison, self-compassion, and predictors of peers and celebrity appearance comparison were collected from 434 college students (Age: Mean = 22; SD = 2.36; Male = Female = 217) in Sialkot, Pakistan. The data was analyzed by using the Hierarchical Regression Model. The results revealed that respondents who compared their appearances to peers and celebrities had increased depression and appearance-based stress while eating constraints didn’t affect the appearance-based comparison, stress, and depression. Moreover, self-compassion significantly moderated the relationship between depression, appearance-based stress, and appearance comparison whereas an insignificant moderation effect is observed between eating restraints and self-compassion. Despite psychological distresses such as depression, appearance-based stress, and eating restraints, appearance comparisons are connected to appearance-based victimization, media appearance pressure, social-cultural appearance pressure, appearance conversation, and self-consciousness.

## 1. Introduction

Experiencing identity concerns about one’s appearance and body is evaluated among adolescents due to alterations stirred in biological and social change, causing adolescents to critically question who they are and how they fit into the world, making it a crucial risk factor for the development of the mental disorder [1–3]. In everyday life, appearance-based stress and body shaming have been linked to adolescents’ self-esteem, appearance-related comments, self-worth, and self-compassion [4]. Researchers have recently focused on examining the link between critical comments and comparisons about one’s appearance and heightened characteristic body shaming [5–7]. It seems logical to assume that individuals who are more concerned about their appearance, self-consciousness, and social comparisons of their appearance are more likely to experience appearance dissatisfaction [4, 8–11]. The most common and easiest way to present themselves and to communicate with other online users in the current digital era is to upload a selfie on appearance-based social network sites (SNSs) like Instagram, Facebook, and others [12]. The positive association between photo-based social network site activities (positive or negative comments, liking or disliking posts) and body shaming has been particularly identified in adolescent girls [13–15]. Recent scientific findings have revealed that college students who view attractive profiles on SNSs, tend to report feeling lower levels of satisfaction with their appearance, this dissatisfaction in turn lead to appearance-based stress and rejection.

### 1.1 Body dissatisfaction impact on health

Body shaming is significantly increased by appearance comparison and negative comments about one’s physical appearance. Moreover, it appears plausible to find strong evidence that individuals who are more self-conscious about their appearance and body shape may suffer more from depression, anxiety, eating disorders, and unhealthy exercise behaviors [3, 16–21]. According to extensive research, body dissatisfaction is substantially connected with body mass index (BMI) [22]. Several studies have demonstrated that appearance-contingent worth positively supports body shape and body surveillance, while negatively affecting appearance self-esteem. Beyond the direct association, appearance-contingent self-worth among adult women moderates the relationship between psychological distress and appearance perfection [23–26]. Cross-sectional studies consistently revealed a connection between body concerns, age, BMI, and psychological dysfunction, however slender body idealization was found to be more prevalent in the mind of young adult women [27–29]. Evidence has revealed that a superficial mismatch between actual and ideal looks and body shape is linked to a lower level of body esteem and increased attention to food constraints, as well as a higher risk of eating disorders among women [30, 31]. The effect of media appearance comparison on body dissatisfaction and eating limitations was justified in a meta-analysis comprising 156 studies [32].

### 1.2 Self-compassion and appearance comparison

Self-compassion involves treating one’s flaws with a balanced viewpoint, kindness, and self-concern rather than self-judgment. Studies have shown that individuals who are able to accept their human experiences and embrace their self-perceptions with self-compassion tend to experience higher levels of life satisfaction, wisdom, happiness, emotional intelligence, and social concern while also experiencing a lower level of sadness and anxiety [20, 33, 34]. The extensive available literature confirms that self-compassion is the crucial form of self-acceptance that leads to fulfillment, whereas negative perceptions of self-compassion lead to severe mental health disorders [35, 36]. Positive attitudes towards one’s appearance are strongly associated with self-esteem and self-compassion, which in turn are negatively associated with psychological distress and positively associated with happiness [33, 37, 38]. Researchers have discovered that dealing with oneself temperately, exaggeration and negative comparison increases the risk of dissatisfaction in individuals, such as self-criticism and unfit attitudes toward self-appearance which lowers self-esteem and increases symptoms of psychological dysfunction [39, 40]. Self-compassion and self-improvement are also found to be inversely connected to shame and low self-esteem, based on cross-sectional data [41–43].

### 1.3 Social network sites and appearance comparison

In recent years, the most prevalent form of self-presentation has been the selfie on social media. The practice allows individuals to interact with communities and friends while also allowing them to assess their body shape and appearance from perspective of others [44–46]. According to self-presentation theory, individuals present themselves as compatible with their ideal self and to please their audience or for self-enhancement. Additionally, users aims to shape their online personas and elicit audience reactions by sharing and manipulating photographs on social media [47, 48]. According to recent studies, the average amount of time spent by individuals on social media each day has increased by 5.2 percent, while uploading selfies on social media is one of the most popular activities among adults [14, 44, 48–50]. Social media interaction with other people through positive and negative comments, likes, and dislikes has increased the risk of psychological negativity and self-dissatisfaction [13, 15, 51, 52]. According to psychological views, appearance comparison on social networks intensifies body shaming, and the existing literature widely supports the role of social media networks in appearance comparison motivation [10, 53, 54]. Many social media users tend to compare their real-life appearance to well-crafted attractive celebrity photographs, mostly they are unaware that these photographs have been finely manipulated, using them as a yardstick to evaluate how closely they match their ideal appearance.

### 1.4 The rationale and hypothesis of the present study

This study focuses on three key objectives. Firstly, it aims to examine whether there is a direct relationship between depression, appearance-based stress, and food restrictions with peer and celebrity appearance comparisons. Secondly, to examine if self-compassion might be used as a potential moderator. Thirdly, to determine whether appearance-based victimization, public self-consciousness, appearance conversation with friends, media appearance pressure, and social culture pressure play any part in the peer and celebrity appearance comparisons.

The study further extends the previous research in two ways: (a) To investigate the influence of appearance comparison on health outcomes such as depression and appearance-based stress in the presence of self-compassion as a moderator. (b) To examine whether there is any significant relationship between appearance comparison and health outcomes in the presence of a moderator; and if there is any, then it would be reasonable to infer that appearance comparison is predicted by social-cultural pressure, media appearance pressure, appearance-based victimization, appearance conversation with friends, and media appearance pressure. As a secondary objective of this study, we examined whether social-cultural pressure would serve as a key conjecturer of peer and celebrity appearance comparison. Additionally, we explored whether negative appearance conversations with friends would be associated with an increase in peer and celebrity appearance comparison and whether public self-consciousness would be linked to peer and celebrity appearance comparison.

## 2. Method

### 2.1. Study design, participants, and procedure

This is a cross-section survey using a stratified random sampling approach with gender as a stratification criterion to pick participants with equal allocation to each gender. A total of 500 BS (Bachelor of Science) students from Govt. Murray Graduate College Sialkot, Pakistan were included in this survey. Students were included in this study whether they had ever been criticized about their appearance in the last two years. The participants took about an hour to complete the questionnaire.

### 2.2. Data collection

The questionnaire was designed to collect data on socio-demographic variables on the following scales relevant to this research.

#### 2.2.1. Appearance-based victimization

We used a modified version of Thompson’s perception short-form scale (e.g., "people made fun of you because of your appearance") to assess perceived verbal victimization. On a 5-point rating scale, 1 = never to 5 = very often, the responses were assessed on six questions intended to assess appearance-related victimizations [55]. In addition, participants were asked whether they had been victimized, and responses (yes = 1)/(no = 0) were recorded.

#### 2.2.2. Appearance-based stress

The perceived appearance-related stress was measured using a modified version of the perceptions of short-form scale in [55]. The responses were recorded on a 5-point rating scale from 1 to 5 on six items designed to assess appearance-related stress (e.g., "People made fun of you because you were heavy: How upset were you?", "People made fun of you because you were frightened to do something: How upset were you?"). The higher scores indicated more stress.

#### 2.2.3. Depression

We used a modified version of the perceptions short-form scale published in [8] to assess depression. On a 4-point rating scale (1 = never, 4 = often), the participant’s responses were recorded for six items of the depression scale (e.g., "I felt unhappy with life or that life was pointless"). The higher scores indicated a high level of depression.

#### 2.2.4. Appearance conversation with friends

On a 5-point rating scale (1 = not at all to 5 = very often), five items of the appearance conversation scale published in [8] were used to measure respondent’s attitudes toward appearance to peers (e.g., "My friend and I talk about how to look attractive, My friends and I talk about what we can do to look our best").

#### 2.2.5. Media-based appearance pressure

On a 5-point scale (1 = strongly disagree to 5 = strongly agree), participant’s media appearance pressure to peers was measured using six items from Thompson’s [56] social-cultural demographic scale; an example item of this scale is "I have to feel pressure from TV and magazines to diet”. This subscale was found to have acceptable reliability and validity [56].

#### 2.2.6. Perceived social culture pressure

Eight items were adopted from Stice and Agras [57] to measure respondents’ social-cultural pressure on peers; two items were eliminated due to their low dependability. Each item is rated on a 5-point scale (1 = not at all, 5 = extremely) and the reliability and validity of the scale have been published in [57], and the modified scale met convergent validity norms.

#### 2.2.7. Eating-restraint

On a 5-point scale (1 = never, 5 = always), three items from the Rosen short-form scale [58] were used to assess respondent’s eating constraint behavior (e.g., "I restrict the amount of food I eat").

#### 2.2.8. Public self-consciousness

Seven items scale was adapted from the Fenigstein short-form scale [59] to assess respondent’s public self-consciousness in front of peers (e.g., "My manner of doing things, I am self-conscious about the way I look"). On a 5-point scale (1 = not at all, 5 = extremely), one item was irrelevant to our culture, therefore it was removed, yet the study met the criterion of convergent validity.

#### 2.2.9. Self-compassion

Self-compassion is the acceptance of one’s flaws and defects, as well as the ability to cope with them with care and kindness [37]. Self-compassion entails treating oneself with kindness, forgiveness, and compassion. On a 5-point scale (1 = almost never, 5 = almost frequently), the items adapted from Filip Roes’s short-form scale [60] were modified to measure respondents’ self-compassion toward their peers.

#### 2.2.10. Celebrity appearance comparison

Six items were adopted from Thompson, Heinberg, and Tantleff-Dunn’s physical comparison scale [61] and were modified to measure respondent’s behavior of appearance comparisons with celebrities on a 5-point scale (1 = never, 5 = always) such as”when I get ready for parties or events, I compare my physical appearance to the physical appearance of celebrities”.

#### 2.2.11. Peer appearance comparison

Three items from Thompson, Heinberg, and Tantleff-Dunn’s physical comparison scale [61] were adopted and reformed to measure respondents frequency of appearance comparisons to peers on a 5-point scale (1 = never, 5 = always): I compare my physical appearance with my peers.

### 2.3 Data analysis

Before conduction of the required statistical analysis to answer the research question, the data was evaluated for any missing observations and outliers. The multivariate Mahalanobis distance (D2) was utilized to find outliers in the data, and the estimated probability was compared at a 5% significance level. The data was then tested for model assumptions such as linearity, independence of observations, normality, and homoscedasticity.

To describe the sample of this study, summary statistics such as mean (standard deviation) for continuous variables, and frequency (percentage) for categorical variables were computed. To assess the correlation among the variables, the Pearson correlation coefficient was used. Moreover, to determine the internal consistency or reliability of various scales such as depression and appearance-based victimization as described in the data collection section, Cronbach’s alpha was used. The effects of appearance comparison on depression, appearance-based stress, and eating constraints with self-compassion as a moderator were assessed in the presence of age and BMI using manual stepwise regression analysis in three separate models. In each of the regression models, the dependent variable was depression, appearance-based stress, or eating constraints. In step one, age and BMI were entered into each model. In steps 2 and 3, the appearance comparisons of peers and celebrities entered, respectively. The moderating influence of self-compassion was assessed in step 4 by adding its interaction with peer appearance comparison and celebrity appearance comparison. In the second set of analyses, we used two separate linear regression models with a manual stepwise approach to examine the relationship between peer and celebrity appearance comparison with social cultural pressure, appearance-based victimization, media appearance pressure, negative appearance conversation with friends, and public self-consciousness adjusted for age and BMI. In step 1, the respondent’s age and BMI were input for each criterion variable. When someone was criticized about their looks, the appearance comparison increased, hence appearance-based victimizations were added to step 2. Step 3 was used to examine the significance of media-based and social-cultural appearance pressure. In step 4, appearance to talk to friends and public self-consciousness was inserted for each regressed variable which revealed how each potential predictor contributed differently to peer and celebrity appearance comparisons.

## 3. Results

**Fig 1:**
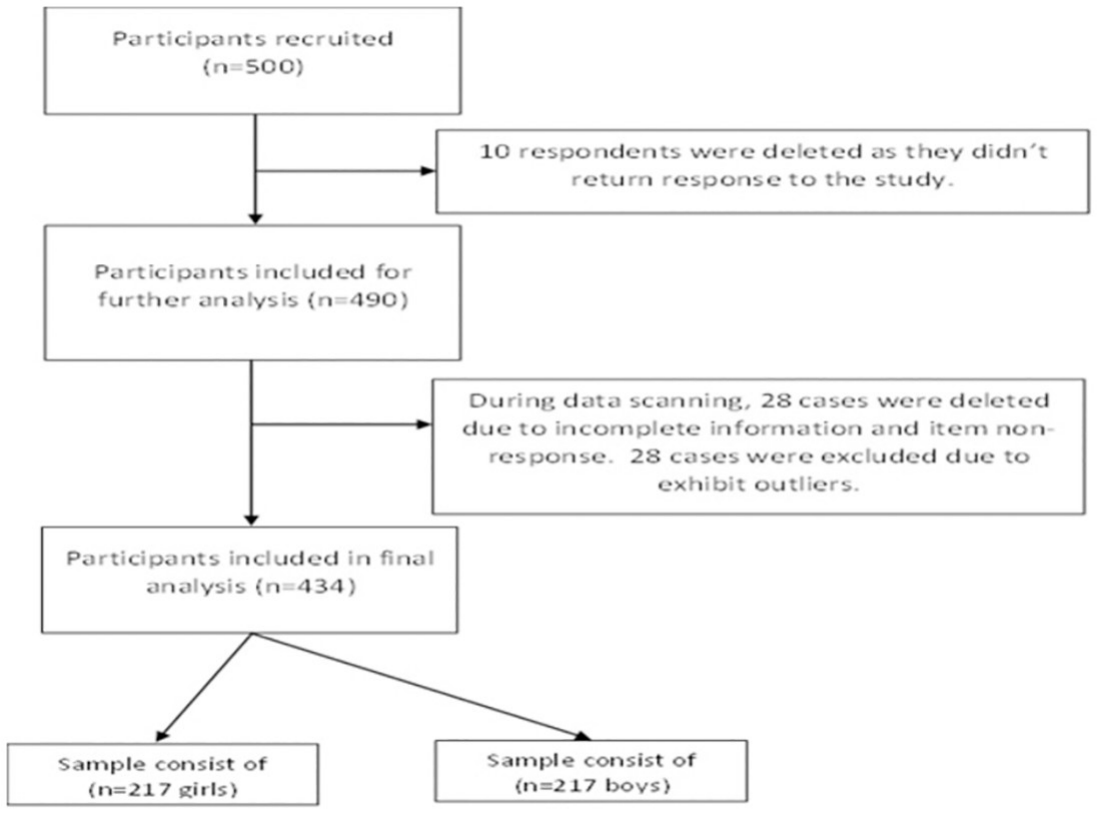
Participants flow diagram.

Initially, 500 students gave consent to participate in the survey; but 10 (2%) did not return the survey’s questionnaire, and 28(5.6%) returned incomplete questionnaires. Twenty-eight of the observations included were outliers and they were affecting the performance of regression models. As a result, these individuals were eliminated from the data set, leaving a final sample size to 434 students, with an equal number of boys and girls (217 each) for the study as shown in Fig 1.

Table 1 shows mean (M), standard error (S.E), standard deviation (S.D), and reliability statistics (Cronbach alpha). The participant’s ages ranged from 18 to 27 years old (M = 22, S.D = 2.36), and their average BMI was 21.02 with S.D = 1.92. Cronbach’s alpha revealed that all the items consistently measure the characteristics, therefore suggesting reliability of the data in our study.

**Table 1:** Characteristics and main outcome variables of the sample (N = 434).

	M ± S. E	S. D	Cronbach alpha
Body mass index	21.02 ± .09	1.92	___
Age	21.64 ± .11	2.36	___
Depression	18.79 ± .25	5.24	0.95
Appearance-based victimization	19.15 ± .28	5.94	0.93
Appearance-based stress	20.57 ± .31	6.47	0.93
Self-compassion	20.07± .32	6.64	0.92
Media appearance pressure	19.30 ± .36	7.60	0.98
Social-cultural pressure	23.68 ± .39	8.15	0.95
Eating constraints	10.73 ± .20	4.08	0.93
Public self-consciousness	16.96 ± .27	5.55	0.92
Appearance conversation with friends	17.41 ± .26	5.53	0.93
Peer appearance comparison	12.75 ± .08	1.70	0.87
Celebrity appearance comparison	20.19 ± .32	6.58	0.92

The Pearson correlation coefficients in Table 2 showed that depression was positively correlated with self-compassion, celebrity appearance-compassion, appearance-victimizations, and appearance-based stress. A similar trend appeared for appearance-based stress. In addition, it was strongly positively correlated with appearance comparison, public self-consciousness, self-compassion, and peer appearance compassion. The findings further revealed that eating constraints had no significant relationship with appearance comparison or appearance conversation, but it had a very weak negative significant relationship with public self-consciousness and media-based pressure (r = -0.116 and r = -0.108, *p*<0.05) respectively. Moreover, there was no evidence of the relationship between self-compassion and appearance compassion, eating constraints, and celebrity appearance comparison. The appearance comparison based on media and peer influences had a substantial positive association with public self-consciousness, social-cultural pressure, and appearance-related victimization, but it had a strong negative relationship with eating constraints. Appearance-compassion and self-compassion were positively correlated (r = 0.280, *p* <0.01). Finally, media-based pressure was correlated with appearance compassion, appearance victimization, and public self-consciousness.

**Table 2:** Pearson correlation among main outcomes and other independent variables.

	**1**	**2**	**3**	**4**	**5**	**6**	**7**	**8**	**9**	**10**	**11**
**1. Depression**	1										
**2. Celebrity-appearance compassion**	.834**	1									
**3. Appearance victimization**	.765**	.722**	1								
**4. Appearance-based stress**	.837**	.913**	.749**	1							
**5. Self-compassion**	.783**	.740**	.718**	.744**	1						
**6. Appearance Conversation**	.834**	.907**	.748**	.990**	.727**	1					
**7. Peer appearance compassion**	.280**	.285**	.265**	.293**	.280**	.279**	1				
**8. Public self-consciousness**	.593**	.633**	.799**	.702**	.537**	.708**	.255**	1			
**9. Eating constraints**	-.013	-.017	-.070	-.033	-.054	-.027	-.078	-.116*	1		
**10. Social cultural pressure**	.551**	.580**	.489**	.598**	.515**	.590**	.506**	.444**	-.090	1	
**11. Media Appearance pressure**	.561**	.577**	.498**	.589**	.514**	.580**	.476**	.454**	-.108*	.976**	1

**Note:** Cases excluded pairwise, N = 434, *p* < .05*, *p* < .01**.

Depression was significantly associated with peer appearance comparison and comparison of celebrity appearances, β (95% CI) = 0.89^a^ (0.61, 1.18) and 0.653^a^ (0.610, 0.696), respectively. The model fit was improved after the inclusion of these two variables (*R*^2^ = 0.70, *p* < 0.001). For appearance-based stress, peer appearance comparison and celebrity appearance comparisons were found to be significant in the model, β (95% CI) = 1.13^a^ (0.78, 1.48) and 0.89^a^ (0.85, 093), and the addition of these two variables, improved the model fit, (*R*^2^ = 0.84, p< 0.001). In the model of eating constraints, only body mass index was significantly adversely associated with eating constraints, β (95% CI) = -0.32^c^ (-0.54, 0.11) and all the other variables including peer appearance comparison and celebrity appearance comparison were not significant. In the case of depression and appearance-based stress, the model fit was further improved by including the interaction of self-compassion with peer and celebrity appearance comparisons, (*R*^2^ = 0.77 and R2 = 0.85) respectively. Hence, self-compassion significantly moderated the above-mentioned associations. The results revealed that the impact on BMI and age were insignificant in the models for depression and appearance-based stress, but they were retained in the model to have adjusted effect estimates (Table 3).

**Table 3:** Results of three separate regression models each assessing the relationship of depression, appearance-based stress, and eating constraints with independent variables in the presence of self-compassion as a moderator.

	Depression*	Appearance-based^Ϯ^stress	Eating constraints^ϯ^
**Independent Variables:**	β (95% CI)	β (95% CI)	β (95% CI)
**Step 1**			
**Age**	.000 (-0.227, 0.227)	.080 (-0.201, 0.361)	.059 (-0.117, 0.234)
**BMI**	-.131 (-0.409, 0.148)	-.062 (-0.405, 0.282)	-.323^**c**^ (-0.538, 0.109)
**Step 2**			
**Peer appearance comparison**	.894^**a**^ (0.612, 1.176)	1.132^**a**^ (0.784, 1.479)	-.156 (-0.383, 0.071)
**Step 3**			
**Celebrity appearance comparison**	.653^**a**^ (0.610, 0.696)	.887^**a**^ (0.848, 0926)	.003 (-0.058, 0.063)
**Step 4**			
**Self-compassion × peer appearance comparison**	.052^**a**^ (0.042, 0.062)	.021^**a**^ (0.011, 0.031)	.001 (-0.016, 0.017)
**Self-compassion × celebrity appearance comparison**	-.023^**a**^(-0.029, -0.017)	-.008^**c**^ (-0.014, -0.002)	-.004 (-0.014, 0.007)

Note: Sample size (N = 434), Depression, appearance-based stress, and eating restraints are dependent variables in the three separate models. β = Regression Coefficient, CI = 95% Confidence intervals upper and lower limit for β, obtained from bootstrapped analyses are represented, p-values for β significance. *p* < 0.001^**a**^, *p* < 0.01^**b**^, *p* < 0.05^**c**^**.** *For the final model of **depression**, R^2^ (Coefficient of determination) = 0.77, and p-value < 0.001 (for the overall model statistical significance).

^**Ϯ**^For the final model of **appearance-based stress**, R^2^ = 0.85, and p-value < 0.001.

^**Ϯ**^For the final model of **eating constraints**, R^2^ = 0.03, and p-value = 0.039

The regression model for celebrity appearance comparison revealed that appearance victimization, social-cultural appearance pressure, and public self-consciousness were significantly positively associated with celebrity appearance comparison; while appearance conversation with friends appeared to be negatively associated with celebrity appearance comparison. The model for peer appearance comparison revealed that appearance victimization and social-cultural appearance pressure were positively associated with peer appearance comparison; while media appearance pressure negatively impacted peer appearance comparison (Table 4).

**Table 4:** Regression models for examining the relationship of peer and celebrity appearance comparison.

	Celebrity appearance comparison^a^	Peer appearance comparison^b^
**Independent variables:**	β (95% CI)	β (95% CI)
**Step 1**		
**Age**	.036 (-0.249, 0.322)	0.032 (-0.042, 0.105)
**BMI**	.055 (-0.295, 0.404)	0.084 (-0.006, 0.173)
**Step 2**		
**appearance victimization**	.805^**c**^ (0.733, 0.878)	0.075^**c**^ (0.049, 0.101)
**Step 3**		
**Media appearance pressure**	-.010 (-0.253, 0.232)	-0.089^**e**^ (-0.172, -0.006)
**Social-cultural pressure**	.248^**d**^ (0.022, 0.473)	0.183^**c**^ (0.105. 0.260)
**Step 4**		
**Appearance conversation with friends**	-.138^**c**^ (-0.218, 0.058)	.020 (-0.023, 0.063)
**Public self-consciousness**	1.00^**c**^ (0.921, 1.078)	-.028 (-0.070, 0.015)

Note: Sample size (N = 434), celebrity appearance comparison, and peer appearance comparison are dependent variables in the two separate models. β = Regression Coefficient, CI = 95% Confidence intervals upper and lower limit for β, obtained from bootstrapped analyses are represented, p-values for β significance. *p* < 0.001^**c**^, *p* < 0.01^**d**^, *or p* < 0.05^**e**^**.**

^**a**^For the final model of **celebrity appearance comparison**, R^2^ (Coefficient of determination) = 0.84, and p-value < 0.001 (for the overall model statistical significance).

^**b**^For the final model of **peer appearance comparison**, R^2^ = 0.28, and p-value < 0.001

## 4. Discussion

The study primarily focused on consolidated models with minimal effort made to demonstrate the impact of peer and celebrity appearance comparison modulated by self-compassion. The prime goal of the present study was to investigate the effect of peer and celebrity appearance comparison on depression, appearance-based stress, and eating restraints, as well as the role of self-compassion as a moderator. The research idea came from the study gap found in prior studies. The above results revealed that peer and celebrity appearance comparison significantly affect the level of depression whereas the role of body mass index and age of respondents do not appear to have a significant influence. Similarly, as the frequency of appearance comparison with celebrities rises, there appears a significant increase in appearance-based stress among respondents. Many studies discovered that young respondents are more conscious about their appearance as compared to elder because there exist a significant relationship between age, body mass index, and the importance given to one’s appearance [41, 62, 63]. The findings suggested that age and body mass index have no influence on participants’ degree of depression because of appearance comparison. Self-comparison with others on appearance or lifestyle leads to self-objectification, a greater proclivity to disapprove of one’s appearance, and significant health issues among individuals. Self-objectification has a negative impact on participants’ self-worth and body esteem, as well as fear of failure and anxiety [64–66]. The current findings are also consistent with previous research, which found that comparing peer looks to celebrity appearance was positively associated with depression [67]. As a result, viewing oneself through the eyes of others has an emotional influence on self-benevolence and it intensifies the amount of self-objectification, which has been identified as the primary cause of depression due to appearance comparison.

**Fig 2:**
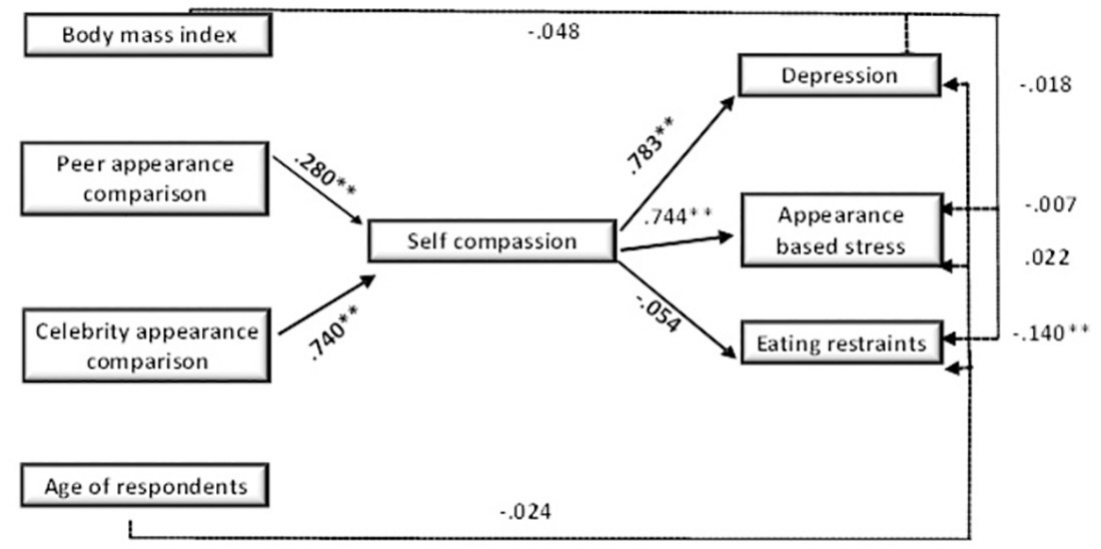
Moderation model illustrating indirect effect of self-compassion on the relationship between health issues and appearance comparison. The numerical values represent correlation coefficients.

Peers, and celebrity appearance comparisons predicted stress owing to apparent appearance (colorism, and body shape). Self-disapproval because of comparing oneself to peers or celebrities causes increased stress, sexual dysfunctions, and is extremely destructive to respondents comfort [68, 69]. Our findings were consistent with the research findings, in which peer and celebrity appearance comparisons were shown to be the significant predictors of appearance-based stress events, while control variables were found to be insignificant in this study. Self-disapproval due to appearance comparisons had previously been established as a major intention for eating disorders or constraints among participants in previous investigations [68, 69]. Moreover, The BMI has a strong negative correlation with dietary limitations, Fig 2. Even though the above-mentioned findings on eating constraints and appearance comparisons were counter to previous studies.

Regarding self-compassion, it was hypothesized that accepting imperfections with kindness rather than reacting with aggression may be thought more secure psychologically in situations where one’s appearance is threatened. Self-judgment or over-identification among individuals was assumed to result in low body esteem, increased restlessness, and a lower sense of well-being about oneself. The analysis revealed that self-compassion positively relates to depression, appearance-based stress, and celebrity appearance comparison, it also moderates the association between depression, appearance-based stress, and appearance comparisons. The lack of moderation by self-compassion was witnessed for eating restraints. The findings aligns with previous research [70–73]. Self-compassion was regarded a more secure response in situations where one’s appearance was threatened especially when faults were accepted with care rather than aggression. As a result, it primes with contentment and acceptance of their looks. According to the findings, positive qualities of self-compassion may maintain self-worth, especially when one’s appearance acceptance is at risk or when individuals perceive their appearance to be below a certain threshold [74].

Although appearance comparison is positively associated with severe health issues, it is more widespread among people in the present era of media, prompting our investigation into potential factors driving the rise in appearance comparisons with peers and celebrities [75, 76].

**Fig 3:**
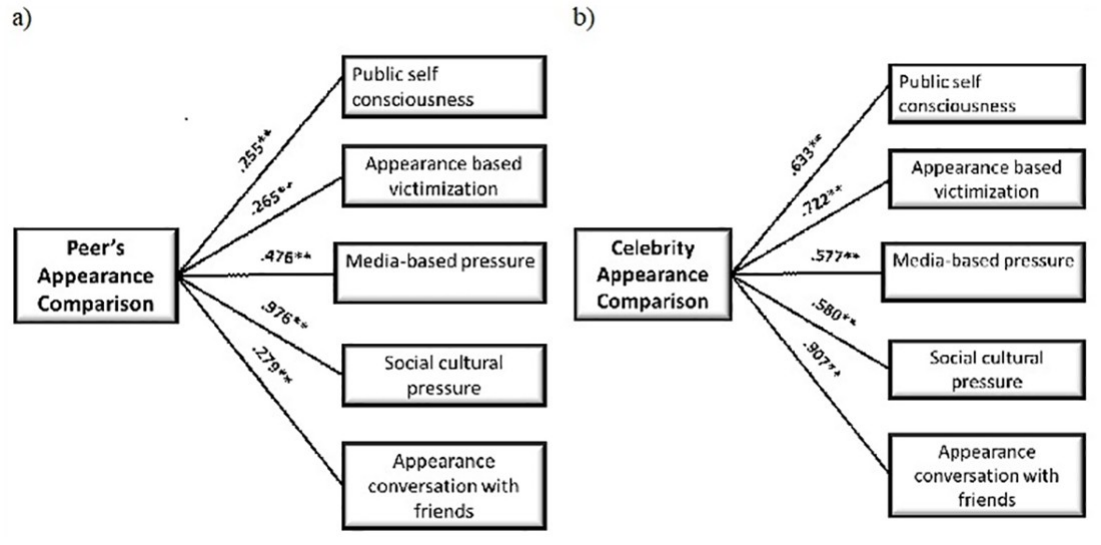
Theoretical framework of the second model: a) appearance comparison with peers and b) appearance comparison with celebrities. The numerical values are correlation coefficients.

The foregoing findings indicate that appearance-based negative comments or victimization are highly associated with appearance compassion. Self-presentation on social media also trigger pessimistic thoughts about one’s appearance, amplifying the respondent’s self-rejection and forcing them to compare themselves to others to change [77, 78]. In literature, young adults habitually compare their appearance with celebrities, and peers body concerns to appearance comparisons with celebrities and peers are boosted by uploading and seeing photographs on social media. The peer and celebrity appearance comparisons are both positively connected to media appearance pressure, Fig 3. The findings are in line with previous studies into the relationship between body shaming and appearance comparison on Facebook, Instagram, and other social media sites [79–84].

The next intriguing hypothesis concerns the link between social-cultural pressure and appearance comparison. In general, social-cultural pressure to change one’s appearance or degree of attractiveness, like ultra-thinness, is conveyed through comments from parents, classmates, and even by seemingly innocuous talk regarding clothes, weight, and body shape. The findings show that comparing one’s looks to that of peers and celebrities is linked to social-cultural standards of attractiveness and societal remarks, Fig 3. According to the findings of the studies, social-cultural pressure contributes to body shaming by giving more importance for unrealistic beauty criteria [8, 85–87]. It has been observed that girls have more appearance-related conversations, such as thinness, body shape, and skin complexion awareness, whereas males tend to be more conscious about muscle gain [88].

Furthermore, the data supported the prediction that appearance conversations with friends contribute to increased self-disapproval and appearance comparison, and that feedback from friends or peers has higher connection with self-judgment and body issues among young people [74, 89–92]. Self-consciousness about appearance happened to be the most important predictor for appearance comparison. Adolescents who were engaged in positive self-representation in front of others focused on photo browsing, photo editing, and fitness, and were more mindful of their looks. The findings corroborate the hypothesis, indicating that self-consciousness was by far the best predictor of celebrities appearances but contrary to peers appearances [93–95].

### 4.1. Limitations and recommendations

In this study several decision domains were considered. First and the foremost, was the frequency with which participants were assessed (state-based comparisons were limited to peers and celebrities) may have an impact on the study’s relationship assessments. The impact of appearance comparisons, for example, may be influenced by an ideal personality in mind or individual appraisal criteria, among other factors. In order to properly capture the above models, future research should compare appearance to ideal personality or establish a baseline for ideal appearance. The study at hand followed the same procedures as the previous studies [4, 41, 96, 97]. Second, the study couldn’t test a theoretical modal and causality due to the use of a cross-sectional design. The significance of the relationship was not recorded and evaluated over time in Pakistan due to cultural differences. Furthermore, future research should compare the cultural effects of different provinces or states on appearance adoption worldwide. Replicating the study in different designs, such as experimental, longitudinal, or comparative contexts, may aid to the discovery of additional potential elements which may contribute to the appearance comparison. Due to the Covid-19 pandemic, the non-response rate was not contacted again. It is also recommended to extend the study to explore the relationship between sexual orientation and body weight dissatisfaction.

### 4.2. Summary

Acceptability of appearance comparison has a high link with depression among participants. Although there was no significant association with eating constraints, the findings demonstrate that appearance comparison contributes to appearance-based stress. It can be concluded that comparing oneself to peers and celebrities can lead to an increase in psychological distress. Depression and appearance-based stress both are moderated by self-compassion while eating restraints, and appearance comparison is not. Furthermore, the study is unique in that it discovered that appearance-based victimization, media-based pressure, social-cultural pressure, appearance discourse with friends, and self-consciousness are potential factors associated with an increase in appearance comparison with peers and celebrities. Self-compassion can be utilized as a control variable for health difficulties, it reduces the threat of one’s appearance approval and its impact on health. It is suggested that respondents do not perceive themselves through the eyes of others or by standards of attractiveness such as thinness, fitness, dieting plan, and so on, which may lead to a reduction in appearance comparison and an increase in self-acceptance happiness.

## References

[1] Bucchianeri M. M., Arikian A. J., Hannan P. J., Eisenberg M. E., & Neumark-Sztainer D. (2013). Body dissatisfaction from adolescence to young adulthood: Findings from a 10-year longitudinal study. Body Image, 10(1), 1–7. doi: 10.1016/j.bodyim.2012.09.001 23084464 PMC3814026

[2] Cash, T. F., & Pruzinsky, T. (2002). Body image: A handbook of theory, research, and clinical practice. Guilford Press.

[3] Griffiths S., Hay P., Mitchison D., Mond J. M., McLean S. A., Rodgers B., et al. (2016). Sex differences in the relationships between body dissatisfaction, quality of life, and psychological distress. *Australian and New Zealand Journal of Public Health*, 40(6), 518–522. doi: 10.1111/1753-6405.12538 27372301

[4] Rogers A., Fuller-Tyszkiewicz M., Lewis V., Krug I., & Richardson B. (2017). A person-by-situation account of why some people more frequently engage in upward appearance comparison behaviors in everyday life. *Behavior Therapy*, 48(1), 19–28. doi: 10.1016/j.beth.2016.09.007 28077218

[5] Lattimore P., & Hutchinson R. (2010). Perceived calorie intake and state body-image satisfaction in women attempting weight loss: A preliminary investigation. *Body Image*, 7(1), 15–21. doi: 10.1016/j.bodyim.2009.08.002 19783237

[6] Fitzsimmons-Craft E. E., Bardone-Cone A. M., Wonderlich S. A., Crosby R. D., Engel S. G., & Bulik C. M. (2015). The relationships among social comparisons, body surveillance, and body dissatisfaction in the natural environment. *Behavior Therapy*, 46(2), 257–271. doi: 10.1016/j.beth.2014.09.006 25645173 PMC8667202

[7] Mills J. S., Minister C., & Samson L. (2022). Enriching sociocultural perspectives on the effects of idealized body norms: Integrating shame, positive body image, and self-compassion. *Frontiers in Psychology*, 13, 983534. doi: 10.3389/fpsyg.2022.983534 36506975 PMC9732395

[8] Jones D. C., Vigfusdottir T. H., & Lee Y. (2004). Body image and the appearance culture among adolescent girls and boys: An examination of friend conversations, peer criticism, appearance magazines, and the internalization of appearance ideals. *Journal of Adolescent Research*, 19(3), 323–339.

[9] O’Brien K. S., Caputi P., Minto R., Peoples G., Hooper C., Kell S., et al. (2009). Upward and downward physical appearance comparisons: Development of scales and examination of predictive qualities. *Body Image*, 6(3), 201–206. doi: 10.1016/j.bodyim.2009.03.003 19447692

[10] Levinson C. A., & Rodebaugh T. L. (2012). Social anxiety and eating disorder comorbidity: The role of negative social evaluation fears. *Eating Behaviors*, 13(1), 27–35. doi: 10.1016/j.eatbeh.2011.11.006 22177392 PMC3244677

[11] Stefano E. C., Hudson D. L., Whisenhunt B. L., Buchanan E. M., & Latner J. D. (2016). Examination of body checking, body image dissatisfaction, and negative affect using ecological momentary assessment. *Eating Behaviors*, 22, 51–54. doi: 10.1016/j.eatbeh.2016.03.026 27086048

[12] Chang L., Li P., Loh R. S. M., & Chua T. H. H. (2019). A study of Singapore adolescent girls’ selfie practices, peer appearance comparisons, and body esteem on Instagram. *Body Image*, 29, 90–99. doi: 10.1016/j.bodyim.2019.03.005 30884385

[13] McLean S. A., Paxton S. J., Wertheim E. H., & Masters J. (2015). Selfies and social media: relationships between self-image editing and photo-investment and body dissatisfaction and dietary restraint. *Journal of Eating Disorders*, 3(1), 1–1.25685349

[14] Ryding F. C., & Kuss D. J. (2020). The use of social networking sites, body image dissatisfaction, and body dysmorphic disorder: A systematic review of psychological research. *Psychology of Popular Media*, 9(4), 41.

[15] Holland G., & Tiggemann M. (2016). A systematic review of the impact of the use of social networking sites on body image and disordered eating outcomes. *Body Image*, 17, 100–110. doi: 10.1016/j.bodyim.2016.02.008 26995158

[16] Cahill S., & Mussap A. J. (2007). Emotional reactions following exposure to idealized bodies predict unhealthy body change attitudes and behaviors in women and men. *Journal of Psychosomatic Research*, 62(6), 631–639. doi: 10.1016/j.jpsychores.2006.11.001 17540220

[17] Dittmar H. (2009). How do “body perfect” ideals in the media have a negative impact on body image and behaviors? Factors and processes related to self and identity. *Journal of Social and Clinical Psychology*, 28(1), 1–8.

[18] Grogan S., Gill S., Brownbridge K., Warnock D., & Armitage C. J. (2016). Women’s long-term reactions to whole-body scanning: A mixed methods approach. *Clothing and Textiles Research Journal*, 34(1), 61–73.

[19] Hargreaves D. A., & Tiggemann M. (2009). Muscular ideal media images and men’s body image: Social comparison processing and individual vulnerability. *Psychology of Men & Masculinity*, 10(2), 109.

[20] Murn L. T., & Steele M. R. (2020). What matters most? Age and gender differences in self-compassion and body attitudes among college students. *Counselling Psychology Quarterly*, 33(4), 541–560.

[21] Barron A. M., Krumrei-Mancuso E. J., & Harriger J. A. (2021). The effects of fitspiration and self-compassion Instagram posts on body image and self-compassion in men and women. *Body Image*, 37, 14–27. doi: 10.1016/j.bodyim.2021.01.003 33556914

[22] Weinberger N. A., Kersting A., Riedel-Heller S. G., & Luck-Sikorski C. (2016). Body dissatisfaction in individuals with obesity compared to normal-weight individuals: a systematic review and meta-analysis. *Obesity Facts*, 9(6), 424–441. doi: 10.1159/000454837 28013298 PMC5644896

[23] Bardone-Cone A. M., Brownstone L. M., Higgins M. K., Fitzsimmons-Craft E. E., & Harney M. B. (2013). Anxiety, appearance contingent self-worth, and appearance conversations with friends about disordered eating: Examining moderator models. *Cognitive Therapy and Research*, 37(5), 953–963.

[24] Manago A. M., Ward L. M., Lemm K. M., Reed L., & Seabrook R. (2015). Facebook involvement, objectified body consciousness, body shame, and sexual assertiveness in college women and men. *Sex Roles*, 72(1), 1–14.

[25] Overstreet N. M., & Quinn D. M. (2012). Contingencies of self-worth and appearance concerns: Do domains of self-worth matter? *Psychology of Women Quarterly*, 36(3), 314–325.

[26] Bardone-Cone A. M., Lin S. L., & Butler R. M. (2017). Perfectionism and contingent self-worth in relation to disordered eating and anxiety. *Behavior Therapy*, 48(3), 380–390. doi: 10.1016/j.beth.2016.05.006 28390500

[27] Stice E. (2002). Sociocultural influences on body image and eating disturbance. *Eating Disorders and Obesity*: *A Comprehensive Handbook*, 2, 103–107.

[28] Pruis T. A., & Janowsky J. S. (2010). Assessment of body image in younger and older women. *The Journal of General Psychology*: *Experimental*, *Psychological*, *and Comparative Psychology*, 137(3), 225–238. doi: 10.1080/00221309.2010.484446 20718224

[29] Runfola C. D., Von Holle A., Trace S. E., Brownley K. A., Hofmeier S. M., Gagne D. A., et al. (2013). Body dissatisfaction in women across the lifespan: Results of the UNC‐SELF and gender and body image (GABI) studies. *European Eating Disorders Review*, 21(1), 52–59. doi: 10.1002/erv.2201 22949165 PMC3745223

[30] Thompson J. K., & Stice E. (2001). Thin-ideal internalization: Mounting evidence for a new risk factor for body-image disturbance and eating pathology. *Current Directions in Psychological Science*, 10(5), 181–183.

[31] Ferguson C. J., Muñoz M. E., Garza A., & Galindo M. (2014). Concurrent and prospective analyses of peer, television, and social media influences on body dissatisfaction, eating disorder symptoms, and life satisfaction in adolescent girls. *Journal of Youth and Adolescence*, 43(1), 1–14. doi: 10.1007/s10964-012-9898-9 23344652

[32] Myers T. A., & Crowther J. H. (2009). Social comparison as a predictor of body dissatisfaction: A meta-analytic review. *Journal of Abnormal Psychology*, 118(4), 683. doi: 10.1037/a0016763 19899839

[33] Neff, K. D. (2008). Self-compassion: Moving beyond the pitfalls of a separate self-concept.

[34] Leary M. R., Tate E. B., Adams C. E., Batts Allen A., & Hancock J. (2007). Self-compassion and reactions to unpleasant self-relevant events: the implications of treating oneself kindly. *Journal of Personality and Social Psychology*, 92(5), 887. doi: 10.1037/0022-3514.92.5.887 17484611

[35] Goodman L. A., Thompson K. M., Weinfurt K., Corl S., Acker P., Mueser K. T., et al. (1999). Reliability of reports of violent victimization and posttraumatic stress disorder among men and women with serious mental illness. *Journal of Traumatic Stress*: *Official Publication of the International Society for Traumatic Stress Studies*, 12(4), 587–599. doi: 10.1023/A:1024708916143 10646178

[36] MacBeth A., & Gumley A. (2012). Exploring compassion: A meta-analysis of the association between self-compassion and psychopathology. *Clinical Psychology Review*, 32(6), 545–552. doi: 10.1016/j.cpr.2012.06.003 22796446

[37] Neff K. D. (2003). The development and validation of a scale to measure self-compassion. *Self and Identity*, 2(3), 223–250.

[38] Neff K. D., & Vonk R. (2009). Self‐compassion versus global self‐esteem: Two different ways of relating to oneself. *Journal of Personality*, 77(1), 23–50. doi: 10.1111/j.1467-6494.2008.00537.x 19076996

[39] Nolen-Hoeksema S., Wisco B. E., & Lyubomirsky S. (2008). Rethinking rumination. *Perspectives on Psychological Science*, 3(5), 400–424. doi: 10.1111/j.1745-6924.2008.00088.x 26158958

[40] Donnelly E., & Kuss D. J. (2016). Depression among users of social networking sites (SNSs): The role of SNS addiction and increased usage. *Journal of Addiction and Preventive Medicine*, 1(2), 107.

[41] Modica C. (2019). Facebook, body esteem, and body surveillance in adult women: The moderating role of self-compassion and appearance-contingent self-worth. *Body Image*, 29, 17–30. doi: 10.1016/j.bodyim.2019.02.002 30818160

[42] Neff K. D., Rude S. S., & Kirkpatrick K. L. (2007). An examination of self-compassion in relation to positive psychological functioning and personality traits. *Journal of Research in Personality*, 41(4), 908–916.

[43] Barnett, M. D., Maciel, I. V., & King, M. A. (2018). Sandbagging and the Self. *Journal of Individual Differences*.

[44] Liu G., Liu Y., Liu A., Li Z., Zheng K., Wang Y., et al. (2018). Context-aware trust network extraction in large-scale trust-oriented social networks. *World Wide Web*, 21(3), 713–738.

[45] Qiu L., Lu J., Yang S., Qu W., & Zhu T. (2015). What does your selfie say about you? *Computers in Human Behavior*, 52, 443–449.

[46] Wu L., Niu G., Ni X., Shao X., & Luo Y. (2019). Body image flexibility moderates the association between photo-related activities on WeChat moments and the body dissatisfaction of female adolescents in China. *Current Psychology*, 1–6.

[47] Baumeister R. F., & Cairns K. J. (1992). Repression and self-presentation: when audiences interfere with self-deceptive strategies. *Journal of Personality and Social Psychology*, 62(5), 851. doi: 10.1037//0022-3514.62.5.851 1593424

[48] Chua T. H. H., & Chang L. (2016). Follow me and like my beautiful selfies: Singapore teenage girls’ engagement in self-presentation and peer comparison on social media. *Computers in Human Behavior*, 55, 190–197.

[49] Lee E. W., Ho S. S., & Lwin M. O. (2017). Explicating problematic social network sites use A review of concepts, theoretical frameworks, and future directions for communication theorizing. *New Media & Society*, 19(2), 308–326.

[50] Kim E., Lee J. A., Sung Y., & Choi S. M. (2016). Predicting selfie-posting behavior on social networking sites: An extension of theory of planned behavior. *Computers in Human Behavior*, 62, 116–123.

[51] Fardouly J., Diedrichs P. C., Vartanian L. R., & Halliwell E. (2015). Social comparisons on social media: The impact of Facebook on young women’s body image concerns and mood. *Body Image*, 13, 38–45. doi: 10.1016/j.bodyim.2014.12.002 25615425

[52] Seekis V., & Kennedy R. (2023). The impact of# beauty and# self-compassion tiktok videos on young women’s appearance shame and anxiety, self-compassion, mood, and comparison processes. *Body Image*, 45, 117–125. doi: 10.1016/j.bodyim.2023.02.006 36870186

[53] Rachel C., Toby N. J., & Amy S. (2018). ‘Selfie’-objectification: The role of selfies in self-objectification and disordered eating in young women. *Computers in Human Behavior*, (79), 68–74.

[54] Meier E. P., & Gray J. (2014). Facebook photo activity associated with body image disturbance in adolescent girls. *Cyberpsychology*, *Behavior*, *and Social Networking*, 17(4), 199–206. doi: 10.1089/cyber.2013.0305 24237288

[55] Thompson J. K., Cattarin J., Fowler B., & Fisher E. (1995). The perception of teasing scale (POTS): A revision and extension of the physical appearance-related teasing scale (PARTS). *Journal of Personality Assessment*, 65(1), 146–157. doi: 10.1207/s15327752jpa6501_11 16367650

[56] Calogero R. M., Davis W. N., & Thompson J. K. (2004). The Sociocultural Attitudes Toward Appearance Questionnaire (SATAQ-3): Reliability and normative comparisons of eating disordered patients. *Body Image*, 1(2), 193–198. doi: 10.1016/j.bodyim.2004.01.004 18089151

[57] Stice E., & Agras W. S. (1998). Predicting onset and cessation of bulimic behaviors during adolescence: A longitudinal grouping analysis. *Behavior Therapy*, 29(2), 257–276.

[58] Rosen J. C., Srebnik D., Saltzberg E., & Wendt S. (1991). Development of a body image avoidance questionnaire. *Psychological Assessment*: *A Journal of Consulting and Clinical Psychology*, 3(1), 32.

[59] Fenigstein A., Scheier M. F., & Buss A. H. (1975). Public and private self-consciousness: Assessment and theory. *Journal of Consulting and Clinical Psychology*, 43(4), 522.

[60] Raes F., Pommier E., Neff K. D., & Van Gucht D. (2011). Construction and factorial validation of a short form of the self‐compassion scale. *Clinical Psychology & Psychotherapy*, 18(3), 250–255. 21584907 10.1002/cpp.702

[61] Thompson J.K., Heinberg L.J., Altabe M.N., & Tantleff-Dunn S. (1999). Exacting beauty: Theory, assessment, and treatment of body image disturbance. Washington, DC: American Psychological Association.

[62] Peat C. M., Peyerl N. L., & Muehlenkamp J. J. (2008). Body image and eating disorders in older adults: a review. *The Journal of General Psychology*, 135(4), 343–358. doi: 10.3200/GENP.135.4.343-358 18959226

[63] Tiggemann M., & Lacey C. (2009). Shopping for clothes: Body satisfaction, appearance investment, and functions of clothing among female shoppers. *Body Image*, 6(4), 285–291. doi: 10.1016/j.bodyim.2009.07.002 19660999

[64] Manago A. M., Graham M. B., Greenfield P. M., & Salimkhan G. (2008). Self-presentation and gender on MySpace. *Journal of Applied Developmental Psychology*, 29(6), 446–458.

[65] Noser A., & Zeigler-Hill V. (2014). Investing in the ideal: Does objectified body consciousness mediate the association between appearance contingent self-worth and appearance self-esteem in women? *Body Image*, 11(2), 119–125. doi: 10.1016/j.bodyim.2013.11.006 24374074

[66] Vandenbosch L., & Eggermont S. (2016). The interrelated roles of mass media and social media in adolescents’ development of an objectified self-concept: A longitudinal study. *Communication Research*, 43(8), 1116–1140.

[67] Peat C. M., & Muehlenkamp J. J. (2011). Self-objectification, disordered eating, and depression: A test of mediational pathways. *Psychology of Women Quarterly*, 35(3), 441–450.

[68] Moradi B., & Huang Y. P. (2008). Objectification theory and psychology of women: A decade of advances and future directions. *Psychology of Women Quarterly*, 32(4), 377–398.

[69] Lindner D., Tantleff-Dunn S., & Jentsch F. (2012). Social comparison and the ‘circle of objectification. *Sex Roles*, 67(3), 222–235.

[70] Ferreira C., Pinto-Gouveia J., & Duarte C. (2013). Self-compassion in the face of shame and body image dissatisfaction: Implications for eating disorders. *Eating Behaviors*, 14(2), 207–210. doi: 10.1016/j.eatbeh.2013.01.005 23557822

[71] Kelly A. C., Vimalakanthan K., & Miller K. E. (2014). Self-compassion moderates the relationship between body mass index and both eating disorder pathology and body image flexibility. *Body Image*, 11(4), 446–453. doi: 10.1016/j.bodyim.2014.07.005 25113286

[72] Homan K. J., & Tylka T. L. (2014). Appearance-based exercise motivation moderates the relationship between exercise frequency and positive body image. *Body Image*, 11(2), 101–108. doi: 10.1016/j.bodyim.2014.01.003 24529336

[73] Adams, S., Mosewich, A., & Polley, S. (2015). Enhancing Well-being–Naturally. *Edited*, 102.

[74] Brophy K., Brähler E., Hinz A., Schmidt S., & Körner A. (2020). The role of self-compassion in the relationship between attachment, depression, and quality of life. *Journal of affective disorders*, 260, 45–52. doi: 10.1016/j.jad.2019.08.066 31493638

[75] Shepperd J. A., Klein W. M., Waters E. A., & Weinstein N. D. (2013). Taking stock of unrealistic optimism. *Perspectives on Psychological Science*, 8(4), 395–411. doi: 10.1177/1745691613485247 26045714 PMC4451212

[76] Leahey T. M., & Crowther J. H. (2008). An ecological momentary assessment of comparison target as a moderator of the effects of appearance-focused social comparisons. *Body Image*, 5(3), 307–311. doi: 10.1016/j.bodyim.2008.03.002 18585108

[77] Fardouly J., & Vartanian L. R. (2016). Social media and body image concerns: Current research and future directions. *Current Opinion in Psychology*, 9, 1–5.

[78] Puccio F., Kalathas F., Fuller-Tyszkiewicz M., & Krug I. (2016). A revised examination of the dual pathway model for bulimic symptoms: The importance of social comparisons made on Facebook and sociotropy. *Computers in Human Behavior*, 65, 142–150.

[79] Mingoia J., Hutchinson A. D., Wilson C., & Gleaves D. H. (2017). The relationship between social networking site use and the internalization of a thin ideal in females: A meta-analytic review. *Frontiers in Psychology*, 8, 1351. doi: 10.3389/fpsyg.2017.01351 28824519 PMC5545754

[80] Lonergan A. R., Bussey K., Mond J., Brown O., Griffiths S., Murray S. B., et al. (2019). Me, my selfie, and I: The relationship between editing and posting selfies and body satisfaction in men and women. *Body image*, 28, 39–43. 30572289 10.1016/j.bodyim.2018.12.001

[81] Stice E., Spangler D., & Agras W. S. (2001). Exposure to media-portrayed thin-ideal images adversely affects vulnerable girls: A longitudinal experiment. *Journal of Social and Clinical Psychology*, 20(3), 270–288.

[82] Grabe S., Ward L. M., & Hyde J. S. (2008). The role of the media in body image concerns among women: a meta-analysis of experimental and correlational studies. *Psychological Bulletin*, 134(3), 460. doi: 10.1037/0033-2909.134.3.460 18444705

[83] Knauss C., Paxton S. J., & Alsaker F. D. (2008). Body dissatisfaction in adolescent boys and girls: Objectified body consciousness, internalization of the media body ideal and perceived pressure from media. *Sex Roles*, 59(9), 633–643.

[84] Tran T. P. T., Vo A. T. N., Nguyen A. H., & Nguyen T. M. (2023). Exploring the Mechanism of Subjective Social Status on Compulsive Shopping Behavior: A Moderated Mediation Model of Self-compassion and Depression. *Journal of Rational-Emotive & Cognitive-Behavior Therapy*, 1–19. doi: 10.1007/s10942-023-00509-y 37360925 PMC10154182

[85] Thompson J. K., Coovert M. D., & Stormer S. M. (1999). Body image, social comparison, and eating disturbance: A covariance structure modeling investigation. *International Journal of Eating Disorders*, 26(1), 43–51. doi: 10.1002/(sici)1098-108x(199907)26:1&lt;43::aid-eat6&gt;3.0.co;2-r 10349583

[86] Vincent M. A., & McCabe M. P. (2000). Gender differences among adolescents in family, and peer influences on body dissatisfaction, weight loss, and binge eating behaviors. *Journal of Youth and Adolescence*, 29(2), 205–221.

[87] Shroff H., & Thompson J. K. (2006). Peer influences, body-image dissatisfaction, eating dysfunction and self-esteem in adolescent girls. *Journal of Health Psychology*, 11(4), 533–551. doi: 10.1177/1359105306065015 16769734

[88] Seekis V., Bradley G. L., & Duffy A. L. (2021). How self-compassion moderates the links between fitspiration use and body concerns in young women. *Mindfulness*, 12(8), 1985–1998.

[89] Shroff H., & Thompson J. K. (2006). Peer influences, body-image dissatisfaction, eating dysfunction and self-esteem in adolescent girls. *Journal of Health Psychology*, 11(4), 533–551. doi: 10.1177/1359105306065015 16769734

[90] Bearman S. K., Presnell K., Martinez E., & Stice E. (2006). The skinny on body dissatisfaction: A longitudinal study of adolescent girls and boys. *Journal of Youth and Adolescence*, 35(2), 217–229. doi: 10.1007/s10964-005-9010-9 16912810 PMC1540456

[91] Jones D. C. (2001). Social comparison and body image: Attractiveness comparisons to models and peers among adolescent girls and boys. *Sex Roles*, 45(9), 645–664.

[92] Ata R. N., Ludden A. B., & Lally M. M. (2007). The effects of gender and family, friend, and media influences on eating behaviors and body image during adolescence. *Journal of Youth and Adolescence*, 36(8), 1024–1037.

[93] Fox J., & Vendemia M. A. (2016). Selective self-presentation and social comparison through photographs on social networking sites. *Cyberpsychology*, *Behavior*, *and Social Networking*, 19(10), 593–600. doi: 10.1089/cyber.2016.0248 27732079

[94] Choukas-Bradley S., Nesi J., Widman L., & Galla B. M. (2020). The appearance-related social media consciousness scale: Development and validation with adolescents. *Body Image*, 33, 164–174. doi: 10.1016/j.bodyim.2020.02.017 32193170

[95] Pila E., Sabiston C. M., Mack D. E., Wilson P. M., Brunet J., Kowalski K. C., et al. (2020). Fitness-and appearance-related self-conscious emotions and sport experiences: A prospective longitudinal investigation among adolescent girls. *Psychology of Sport and Exercise*, 47, 101641.

[96] Leanne Dillard S., Smith S. R., & Hancock D. W. (2019). Variability of ergovaline and total ergot alkaloid expression among endophytic tall fescue cultivars. *Crop Science*, 59(6), 2866–2875.

[97] Boursier V., Gioia F., & Griffiths M. D. (2020). The objectified body consciousness, body image control in photos, and problematic social networking: the role of appearance control beliefs. *Frontiers in Psychology*, 11. 10.3389/fpsyg.2020.00147 PMC7052303 32158409

